# Enhancing Solubility and Bioefficacy of Stilbenes by Liposomal Encapsulation—The Case of Macasiamenene F

**DOI:** 10.1021/acsomega.3c07380

**Published:** 2024-02-15

**Authors:** Veronika Brezani, Nicolas Blondeau, Jan Kotouček, Eva Klásková, Karel Šmejkal, Jan Hošek, Eliška Mašková, Pavel Kulich, Vilailak Prachyawarakorn, Catherine Heurteaux, Josef Mašek

**Affiliations:** † Department of Molecular Pharmacy, Faculty of Pharmacy, Masaryk University, Palackého tř. 1946/1, CZ-612 00 Brno, Czech Republic; ‡ Department of Pharmacology and Toxicology, Veterinary Research Institute, Hudcova 296/70, CZ-621 00 Brno, Czech Republic; § IPMC, UMR 7275, Université Côte d’Azur, CNRS, 660 Route des Lucioles, Sophia Antipolis, F-06560 Valbonne, France; ∥ Department of Pharmacology, Faculty of Medicine, Masaryk University, Kamenice 753/5, CZ-625 00 Brno, Czech Republic; ⊥ Department of Natural Drugs, Faculty of Pharmacy, Masaryk University, Palackého tř. 1946/1, CZ-612 00 Brno, Czech Republic; # Chulabhorn Research Institute, Kamphaeng Phet 6 Road, Laksi, TH-10210 Bangkok, Thailand

## Abstract

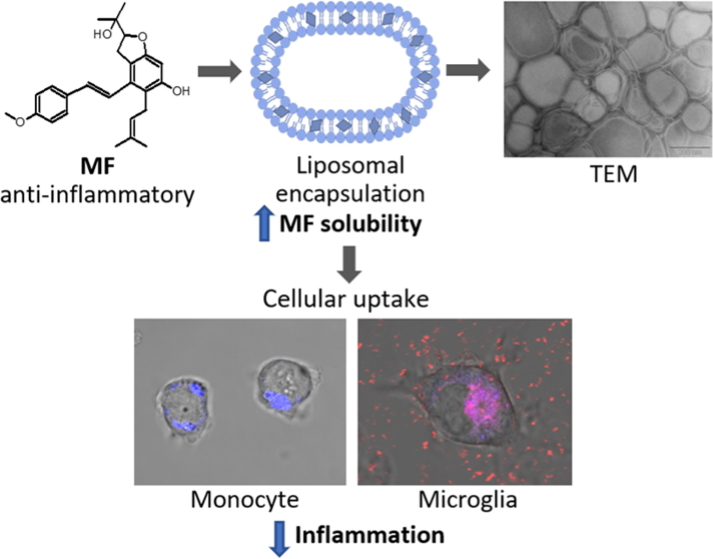

Stilbenes in food and medicinal plants have been described as potent antiphlogistic and antioxidant compounds, and therefore, they present an interesting potential for the development of dietary supplements. Among them, macasiamenene F (MF) has recently been shown to be an effective anti-inflammatory and cytoprotective agent that dampens peripheral and CNS inflammation *in vitro*. Nevertheless, this promising molecule, like other stilbenes and a large percentage of drugs under development, faces poor water solubility, which results in trickier *in vivo* administration and low bioavailability. With the aim of improving MF solubility and developing a form optimized for *in vivo* administration, eight types of conventional liposomal nanocarriers and one type of PEGylated liposomes were formulated and characterized. In order to select the appropriate form of MF encapsulation, the safety of MF liposomal formulations was evaluated on THP-1 and THP-1-XBlue-MD2-CD14 monocytes, BV-2 microglia, and primary cortical neurons in culture. Furthermore, the cellular uptake of liposomes and the effect of encapsulation on MF anti-inflammatory effectiveness were evaluated on THP-1-XBlue-MD2-CD14 monocytes and BV-2 microglia. MF (5 mol %) encapsulated in PEGylated liposomes with an average size of 160 nm and polydispersity index of 0.122 was stable, safe, and the most promising form of MF encapsulation keeping its cytoprotective and anti-inflammatory properties.

## Introduction

Solubility of a drug is one of the essential factors to be considered not only for oral but also for parenteral administration, enabling the drug to achieve the desired concentration in the systemic circulation and obtain the required pharmacological response at the target site.^1^ However, more than 40% of new chemical entities developed in the pharmaceutical industry face water insolubility, which is often a cause of their low bioavailability.^2^ This is also the case in preclinical assays for many natural polyphenol compounds where bioavailability is limited by low solubility in biological fluids and fast metabolization *in vivo*.^3^ Improving solubility requires some physical and/or chemical modifications of drugs, such as micronization, preparation of nanosuspension, salt formation, or complexation.^2^ Encapsulation into liposomes is a formulation strategy that can enhance the solubility^4^ and bioefficacy of small molecules exhibiting poor biodistribution or pharmacokinetics when administered alone.^5^ Size, composition, lamellarity, and charge of liposomes can be variously adapted according to the target of their application. Moreover, coupling with a specific antibody or ligands may enable site-specific delivery.^6^ Several liposomal formulations of anticancer drugs have already been marketed, and other liposomal formulations have recently found applications, such as SARS-CoV-2 mRNA vaccine carriers. Thanks to their numerous advantages, liposomes have also been proposed as a future drug delivery system to the CNS for neurological disorders,^7^ including acute cerebral pathologies such as stroke.^8^ Among various types of nanoparticles, liposomes have been extensively studied due to their low toxicity, biocompatibility, and biodegradability. Moreover, they present several advantages, such as improved stability and protection of the drug from degradation in a biological environment, increased circulation time in the bloodstream, and accumulation in the target tissue.^9^ Liposomes are also able to attenuate the toxicity of the encapsulated therapeutic agent.^10,11^ Prolonged systemic circulation and extended liposomal half-time can be easily obtained by surface modification with poly(ethylene glycol) (PEG) that masks the recognition by opsonins and reduces clearance by the reticuloendothelial system of macrophages.^12^ Altogether, the liposomal strategy leads to enhanced efficacy of drugs and improved therapeutic index.^13^

Stilbenoids are phytochemicals naturally occurring in food and medicinal plants, of which *trans*-resveratrol (RSV) is the most known representative. We and others demonstrated that stilbenoids contained in foods, such as different edible berries, grapes, peanuts, rhubarb, and beverages like red wine or white tea, possess *in vitro* anti-inflammatory,^14,15^ antioxidant,^16^ cardio-,^17^ neuro-,^18^ and cytoprotective properties,^19^ but a poor solubility in aqueous solutions complicates their administration *in vivo* and decreases their bioavailability.^20^ Because the RSV hydrosolubility is approximately 0.03 mg mL^–1^, it is classified as “practically insoluble” in water according to the European and U.S. Pharmacopeia definition.^21,22^ Moreover, fast metabolization described for RSV and some derivatives contributes to its poor bioavailability.^21^ In our previous studies, we described a potent *in vitro* anti-inflammatory potential of prenylated^23,24^ and nonprenylated stilbenes.^15^ Among them, macasiamenene F (MF), a natural prenylated stilbene from *Macaranga siamensis* S.J. Davies exhibits the ability to dampen better than prednisone the activity of transcription factors nuclear factor κB (NF-κB) and activator protein-1 (AP-1) in THP-1-Xblue-MD2-CD14 monocytes, prevents IκBα degradation, and reduces downstream TNF-α and IL-1β secretion in THP-1 human macrophages. Moreover, MF exerts anti-inflammatory effects also at the CNS level by inhibiting the gene and protein expression of TNF-α and IL-1β in lipopolysaccharide (LPS)-challenged mouse microglia, whatever the treatment paradigms (pre-, co-, and post-treatment). Furthermore, MF displays cytoprotective effects against LPS-induced loss of microglia.^24^ To optimize MF effects *in vivo* excluding the use of chemical vehicles, an appropriate liposomal MF formulation was designed to overcome poor MF hydrosolubility in such a way that encapsulation could be transferable for other promising stilbenoid compounds. Nine MF liposomal formulations with a size ranging from 100 to 200 nm, differing in the lipid composition and surface charge were designed. Then, the obtained formulations were tested in the *in vitro* assays on the models of systemic or CNS inflammation for identifying the safest formulation, which keeps the anti-inflammatory effects of MF unencapsulated.

## Results and Discussion

### Improvement of MF Solubility by Encapsulation into Liposomes

**Figure 1:**
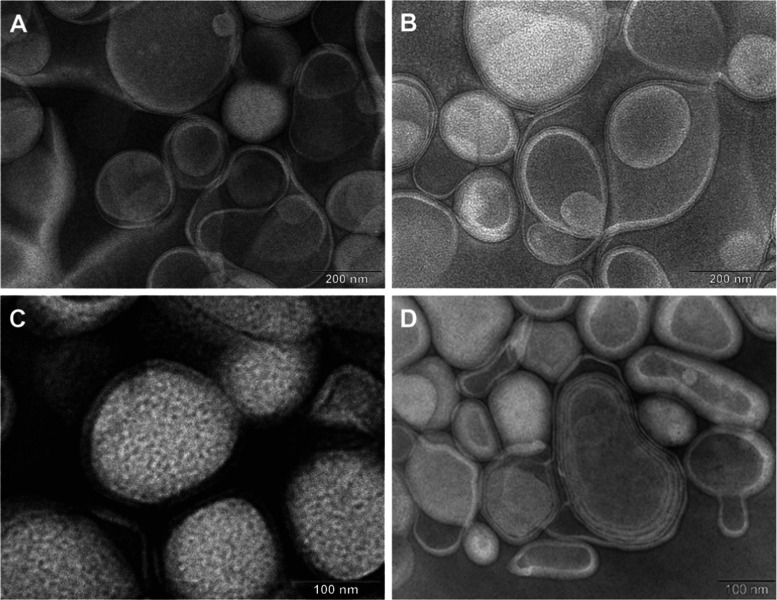
TEM micrographs of MF liposomal formulations. Morphological representation of liposomal types: (A) anionic liposomes (sample II); (B) neutral liposomes (sample IV); (C) PEGylated liposomes (sample PEG-VIII); and (D) cationic liposomes (sample VI). Scale bars—200 nm (A, B), 100 nm (C, D).

To find an appropriate MF liposomal formulation, eight types of conventional liposomes **I–VIII** differing in ζ-potential determining their surface charge being in contact with the aqueous solution were prepared. The size and composition of formulations were selected after a detailed literature review for parenteral application with a preferred delivery of drug to the brain and its further testing on rodent models of neurological disorders with a strong inflammatory component, such as Alzheimer’s disease (AD), Parkinson’s disease (PD), or stroke.^25−27^ To achieve efficient delivery of agents to the brain through the blood–brain barrier (BBB), the size of liposomal nanoparticles is one of the important factors to consider.^25,26^ Previous studies on rodent models of ischemic stroke demonstrated that liposomes < 200 nm are able to reach brain and accumulate in the ischemia/reperfusion (I/R) region when administered intravenously.^28−30^ Liposomes of size < 200 nm have also attracted interest in rodent models of AD.^31^ The liposomal surface charge is another predominant factor of a liposomal formulation determining the fate of nanoparticles *in vivo* and the passage of nanoparticles and any encapsulated content into cells.^32^ Thereby, we evaluated several types of liposomal formulations: anionic (**I**–**III**) with ζ-potential ranging from −28.1 to −14.6 mV, neutral (**IV**; –2.4 mV), and cationic (**V**–**VIII**; 20.1–31.9 mV). The composition of **VIII** was later modified with PEG (**PEG**-**VIII**) (Table 1) so that PEGylation of nanoparticles prolongs their circulation time in the body, prevents opsonization and subsequent clearance of liposomes, and improves systemic and brain delivery.^27,33^ The content of MF incorporated representing five molar percent was a predetermined formulation meeting the criteria for size (<200 nm) and homogeneity (PDI < 0.3) (Table S1), as we previously shown for other compounds.^34,35^ Liposomal encapsulation has also been shown effective in case of RSV (0.3–11 mol %)^36^ and its dimer trans-ε-viniferin.^37^ The morphological characteristics and homogeneity of the liposomal formulations were investigated through transmission electron microscopy (TEM) and dynamic light scattering (DLS) as two commonly used methods to study the size of nanoparticles.^38,39^Figure 1 displays representative TEM images of anionic, cationic, neutral, and PEGylated liposomes corresponding to the formulations outlined in Table 1. These images collectively illustrate the structural features of each liposomal type. The DLS analysis revealed a notable homogeneity and the absence of aggregation or destruction in the liposomal structures, both in the presence and absence of the active compound. Detailed distribution profiles for intensity, volume, and number are provided in Figures S1–S4. These metrics provide a detailed overview of the size distribution within each liposomal population. The TEM images further corroborate the findings of DLS analyses, demonstrating a consistent and uniform morphology across all liposomal formulations.

**Table 1:** Experimental Samples of MF Liposomal Formulations **I**–**VIII**, **PEG-VIII**, and Their Reference Controls **C/I**–**C/VIII** and **C/PEG-VIII** without MF^a^

type	sample name	type of liposome	composition (mol %)	*Z*-average (nm)	PDI	ζ-potential (mV)
experimental samples	**I**	anionic	30% POPG, 5% MF, 65% EPC	138 ± 6	0.143 ± 0.005	–28.1 ± 1.1
	**II**	anionic	20% POPG, 5% MF, 75% EPC	157 ± 10	0.222 ± 0.040	–23.2 ± 1.6
	**III**	anionic	10% POPG, 5% MF, 85% EPC	140 ± 6	0.149 ± 0.037	–14.6 ± 2.1
	**IV**	neutral	5% MF, 95% EPC	188 ± 8	0.104 ± 0.058	–2.4 ± 0.7
	**V**	cationic	10% DC-CHOL, 5% MF, 85% EPC	167 ± 7	0.134 ± 0.014	20.1 ± 1.7
	**VI**	cationic	15% DC-CHOL, 5% MF, 80% EPC	161 ± 5	0.106 ± 0.015	22.7 ± 2.0
	**VII**	cationic	20% DC-CHOL, 5% MF, 75% EPC	159 ± 7	0.135 ± 0.028	25.9 ± 2.2
	**VIII**	cationic combined	15% DC-CHOL, 25% CHOL, 5% MF, 55% EPC	168 ± 3	0.080 ± 0.006	31.9 ± 2.8
	**PEG-VIII**	PEGylated VIII	15% DC-CHOL, 25% CHOL, 5% MF, 50% EPC + 5% DSPE-PEG 2000	160 ± 9	0.122 ± 0.038	0.2 ± 0.6
reference/control samples	**C/I**	anionic	30% POPG, 70% EPC	124 ± 4	0.096 ± 0.007	–26.9 ± 1.8
	**C/II**	anionic	20% POPG, 80% EPC	125 ± 5	0.131 ± 0.018	–20.9 ± 2.2
	**C/III**	anionic	10% POPG, 90% EPC	132 ± 7	0.146 ± 0.020	–14.4 ± 1.6
	**C/IV**	neutral	100% EPC	194 ± 12	0.188 ± 0.079	–1.61 ± 1.8
	**C/V**	cationic	10% DC-CHOL, 90% EPC	147 ± 6	0.133 ± 0.005	19.5 ± 1.5
	**C/VI**	cationic	15% DC-CHOL, 85% EPC	138 ± 5	0.142 ± 0.010	21.3 ± 2.5
	**C/VII**	cationic	20% DC-CHOL, 80% EPC	137 ± 6	0.145 ± 0.008	25.7 ± 1.3
	**C/VIII**	cationic combined	15% DC-CHOL, 25% CHOL, 60% EPC	166 ± 7	0.135 ± 0.011	27.9 ± 0.6
	**C/PEG-VIII**	PEGylated VIII	15% DC-CHOL, 25% CHOL, 55% EPC + 5% DSPE-PEG 2000	156 ± 4	0.169 ± 0.058	0.2 ± 0.1

a The % of used lipids represents the percent ratios of their molar concentrations. PDI = polydispersity index. MF—macasiamenene F, DC–CHOL —3β-[*N*-(*N*′,*N*′-dimethylaminoethane)-carbamoyl]cholesterol hydrochloride, DSPE-PEG 2000—1,2-distearoyl-*sn*-glycero-3-phosphoethanolamine-*N*-[amino(polyethylene glycol)-2000], EPC—1,2-dioleoyl-*sn*-glycero-3-ethylphosphocholine (chloride salt), POPG—1-palmitoyl-2-oleoyl-*sn*-glycero-3-phospho-(1′-*rac*-glycerol) (sodium salt). Size, polydispersity index, and ζ-potential are reported as mean ± standard deviation (*n* = 3).

### Transition of MF into a Model of the Lipid Bilayer and the Cellular Uptake

**Figure 2:**
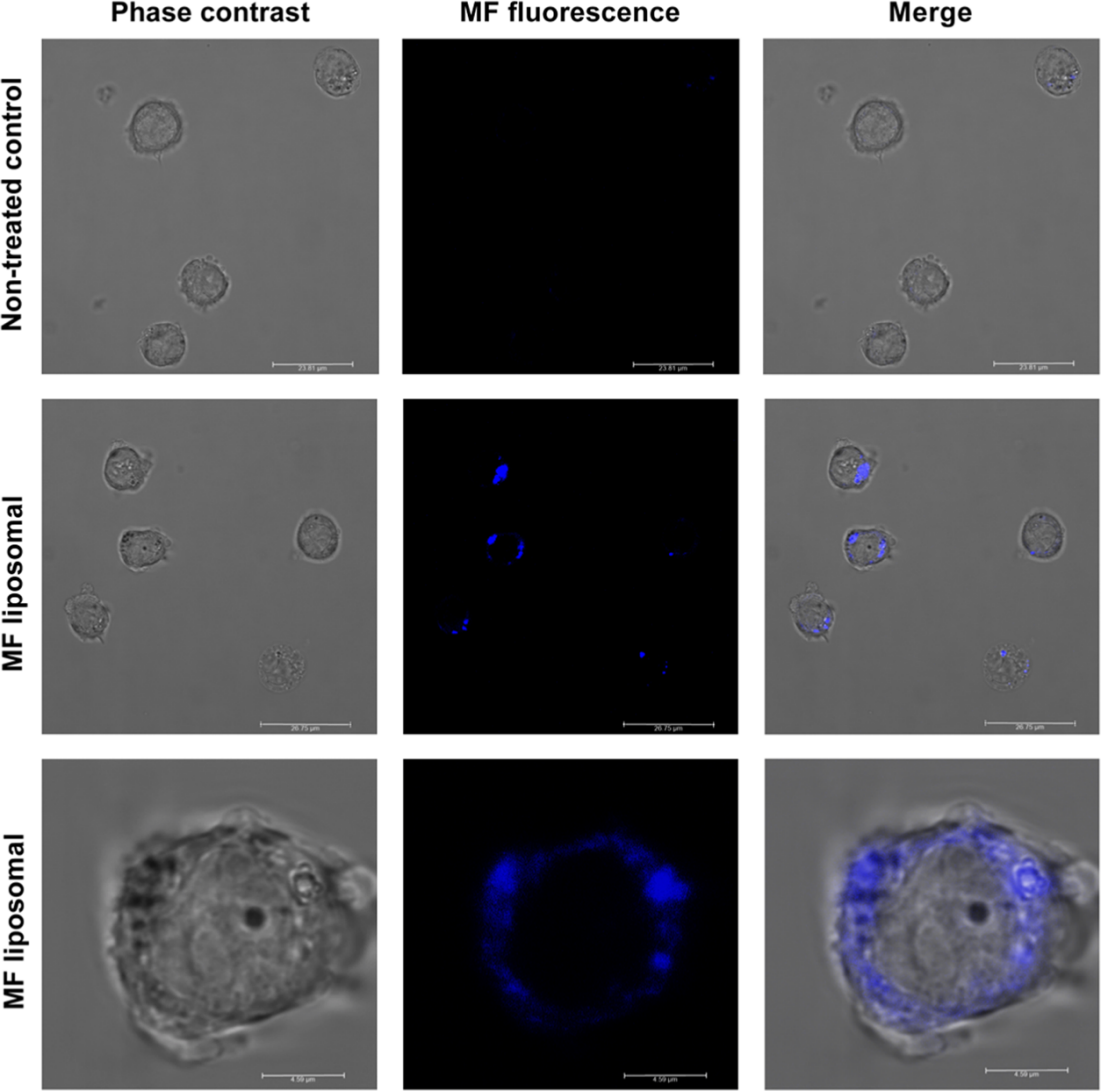
MF liposomal uptake by THP-1-XBlue-MD2-CD14 cells. The uptake of MF liposomal VI by THP-1-XBlue-MD2-CD14 human monocytes at 24 h was detected by live cell imaging. Scale bars: 24 μm (upper row), 27 μm (middle row), and 4.6 μm (lower row).

To estimate the efficiency of MF encapsulation and MF liposomal incorporation into cells, we used the characteristics of stilbenes to be fluorescent under UV light, emitting violet-blue fluorescence to localize them *in vivo*.^40^ From the fluorescence spectral analysis, the excitation maximum (309 nm) and emission maximum (422 nm) were determined (Figure S5). The obtained fluorescence results, as illustrated in Figure S6, enabled a quantitative assessment of the efficiency of MF encapsulation within the liposomes. Stilbenes, based on their lipophilic character showed high encapsulation efficiency into liposomes up to 98% as has been demonstrated for the reference stilbene resveratrol in phosphatidylcholine-cholesterol liposomes.^41^ The lipophilicity of phenolics further increases with methoxy-^42^ and prenyl-^43^ groups as shown for resveratrol-methoxylated derivative pterostilbene.^44^ Furthermore, the fluorescence results contribute to our understanding of the uptake dynamics of MF-loaded liposomes by monocytes.^45^ The distinctive fluorescence signals associated with liposomal formulations aided in tracking the cellular internalization of MF, providing valuable information about the interaction between liposomes and monocytes (Figures 2 and S7B).

To monitor the incorporation of MF into liposomes, we used the model of the lipid bilayer membrane represented by EPC liposomes in water (38 μM). We measured the fluorescence intensity of EPC lipids and MF (2 μM) aqueous dispersion as well as the mixture of both in a ratio of 1:1 where MF represented 5 mol % compared to EPC, similarly as for the prepared liposomal formulations. We observed no increase in the fluorescence signal of MF aqueous dispersion compared to EPC lipids, which confirms that MF, similar to other stilbenes, is practically insoluble in water. When MF aqueous dispersion was mixed with EPC liposomes, the fluorescence intensity increased immediately (*t* = 0) (Figure S7A). The emission maximum wavelength was in accordance with that observed for MF dissolved in ethanol (Figure S5), confirming the rapid incorporation of this lipophilic compound in the lipid bilayer.

### Liposomal Encapsulation Decreases the Toxicity of MF on Human Monocytes

**Figure 3:**
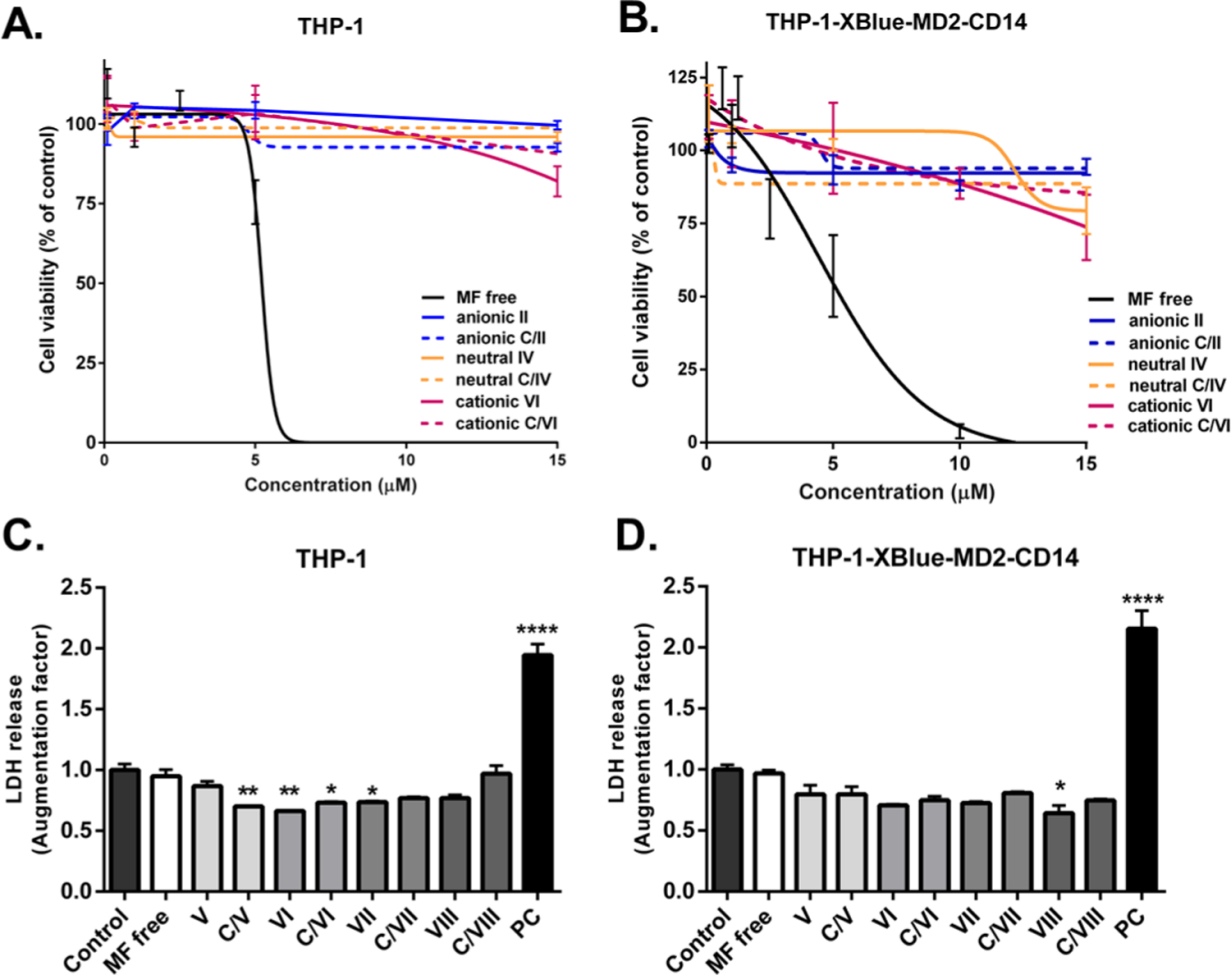
Effect of MF liposomes on the cell viability of THP-1 and THP-1-XBlue-MD2-CD14 monocytes. Effect of anionic (II), neutral (IV), and cationic (VI) MF liposomal and their respective controls **C/II**, **C/IV**, and **C/VI** on the cell viability of (A) THP-1 cells and (B) THP-1-Xblue-MD2-CD14 monocytes corresponding to metabolically active cells determined by a WST-1 assay. (C) Effect of cationic MF liposomal **V**–**VIII** (1 μM) and respective controls (**C/V**–**C/VIII**) on LDH release in THP-1 cells and (D) THP-1-Xblue-MD2-CD14 monocytes. *P < 0.05, **P < 0.01 compared to control (spontaneous release). PC—positive control (Triton 2%). Mean ± SEM, *N* = 3. One-way ANOVA with Bonferroni’s post hoc test.

Since the safety of the drug is one of the most important factors, we first evaluated the dose–response effect of the prepared MF liposomal formulations at concentrations of MF in the range of 0.1–15 μM and their respective liposomal controls at the same concentrations of lipids on LDH release and viability of THP-1 and THP-1-Xlue-MD2-CD14 monocytes, as we previously showed MF toxicity above that concentration in this model.^24^ Interestingly, neither the liposomal MF formulations nor their respective controls without MF significantly altered the viability of THP-1 and THP-1-XBlue-MD2-CD14 monocytes. Their IC_50_ values were over 5 μM for both cell lines, as displayed in Figure 3A,B for one representative of each group. The IC_50_ values of all formulations can be found in Table S2. Considering that IC_50_ of MF free is 5.7 ± 1.1 μM for THP-1 cells and 5.4 ± 1.3 μM for THP-1-XBlue-MD2-CD14 cells,^45^ these results indicated that the incorporation of MF into liposomes is associated with a decreased toxicity risk in line with what has been observed for other encapsulated compounds^46^ and commercially available drugs.^12^ Its relative safety was previously described also in other cell lines, including A549, HepG2, HuCCA-1, and MOLT-3 cells.^47^ Moreover, cationic formulations **V**–**VIII** at concentrations of 1 μM of MF encapsulated, as well as liposomes alone, attenuated the basal LDH release in THP-1 cells up to 30% (Figure 3C) and up to 36% in THP-1-XBlue-MD2-CD14 cells (Figure 3D).

### Liposomal Formulations Reduce NF-κB/AP-1 Activity in THP-1-XBlue-MD2-CD14 Human Monocytes

**Figure 4:**
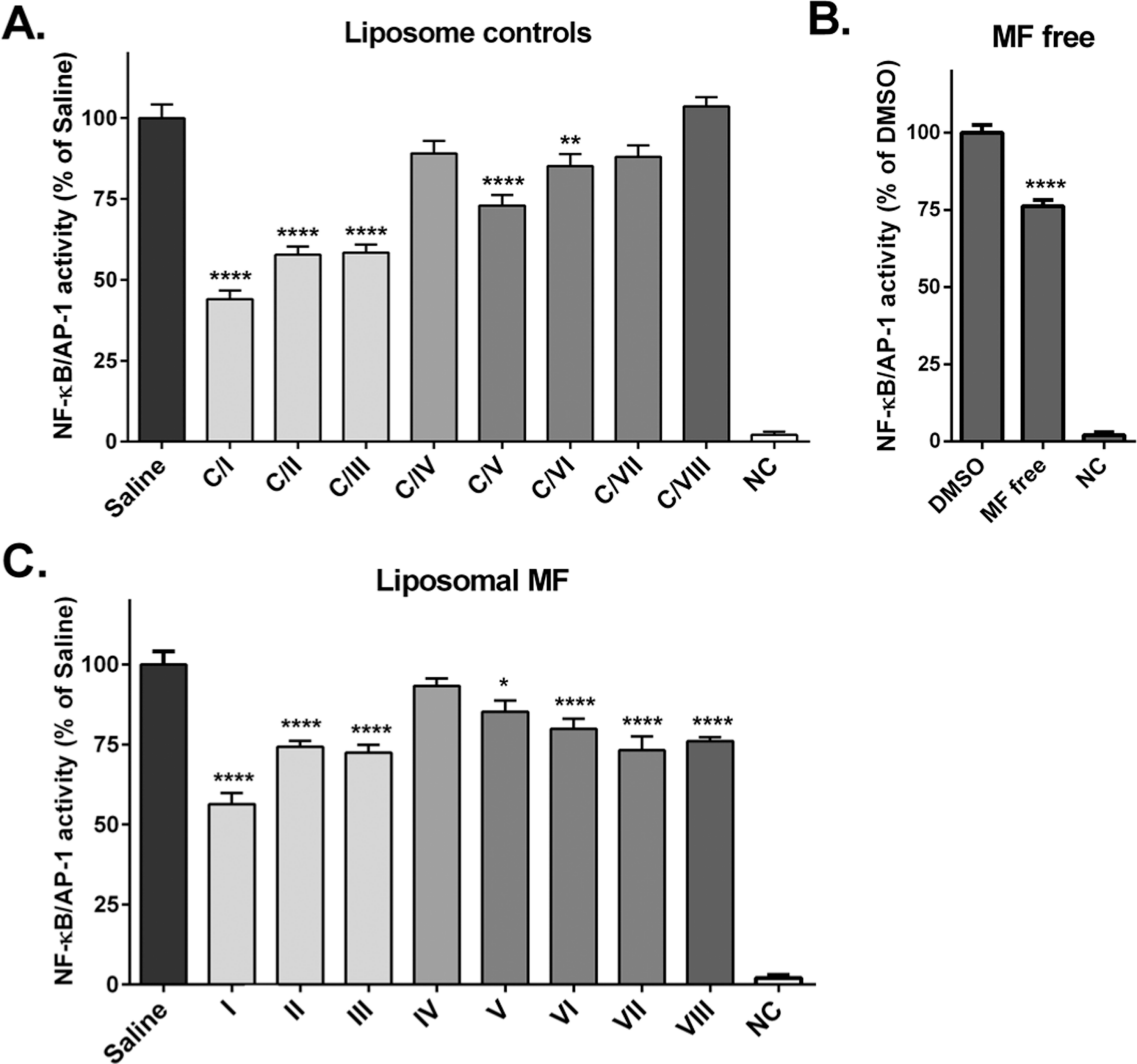
Effect of liposomal controls and MF liposomal formulation **I**–**VIII** on NF-κB/AP-1 activity in THP-1-XBlue-MD2-CD14 monocytes. (A) Effect of 1 h pretreatment by liposomal controls **C/I**–**C/VIII** (without MF) on LPS-stimulated NF-κB/AP-1 activity detected after 24 h. (B) Effect of 1 μM MF free pretreatment on LPS-stimulated NF-κB/AP-1 activity at 24 h compared to the vehicle (0.1% DMSO). (C) Effect of MF liposomal **I**–**VIII** (1 μM) pretreatment on LPS-stimulated NF-κB/AP-1 activity at 24 h compared to saline. NC—negative control (LPS-nonstimulated). *P < 0.05, **P < 0.01, ****P < 0.0001 compared to respective control/vehicle. Mean ± SEM, *N* = 3. One-way ANOVA with Bonferroni’s post hoc test.

Since we previously described that nonencapsulated MF dampens LPS-induced activation of the TLR4/NF-κB pathway^24^ at a pharmacologically and clinically relevant concentration of 1 μM, we investigated the effect of liposomal encapsulation on this important anti-inflammatory feature. Hence, we performed a head-to-head comparison of the effects of MF liposomal **I**–**VIII** at a concentration of 1 μM, together with the effect of their respective controls **C/I**–**C/VIII** at the same concentration of lipids on the NF-κB/AP-1 activity in THP-1-XBlue-MD2-CD14 cells. Surprisingly, anionic formulations **C/I**–**C/III** and cationic **C/V–VI** displayed by themselves a significant inhibitory activity of NF-κB/AP-1 being in accord with previous studies.^48,49^ This inhibitory effect of liposomes themselves may be attributed to different phospholipid contents as the anti-inflammatory potential of some anionic phospholipids such as POPG^48^ and cationic ones^50^ has been previously reported. On the contrary, neutral liposomes (**C/IV**) and cationic ones with higher ζ-potential (**C/VII**–**C/VIII**) alone did not show any significant effect on the NF-κB/AP-1 activity (Figure 4A). MF liposomal formulations **I**–**III** and **V** showed a lower effect compared to their respective controls **C/I**–**III** and **C/V**. Neutral MF liposomal (**IV**) formulation did not exert any significant effect, and cationic formulations **VI**–**VIII** displayed the NF-κB/AP-1 inhibitory potential ranging from 20 to 27% (Figure 4C) similar to that of free MF (Figure 4B). When comparing the effects of MF liposomal formulations to their respective lipid controls, the greatest inhibitory effect was achieved by MF liposomal **VIII**.^45^ Overall, these results demonstrate how the composition and surface charge influence the effect of the encapsulated molecule, and the selection of the appropriate composition is a highly important factor to be considered. Based on these observations, formulation **VIII** displaying a safe character and similar inhibitory effect of NF-κB/AP-1 than free MF was selected for the studies at CNS level. Since PEGylation could significantly prolong liposomal cargo half-life by steric stabilization and increase the chances for brain delivery, we modified the composition of MF liposomal **VIII** using PEG. PEGylated cationic liposomes with similar composition and size have been previously described as a promising way of drug delivery through the BBB for the treatment of neurological disorders such as stroke.^8,25^ We therefore evaluated the interest of this type of liposomes in *in vivo* assays, where activated microglia play an important role in the inflammatory response and progression of brain disorders.

### Anti-Inflammatory Dose of MF Liposomal Does Not Alter the Viability and Metabolic Activity of BV-2 Mouse Microglia and Primary Cortical Neurons

**Figure 5:**
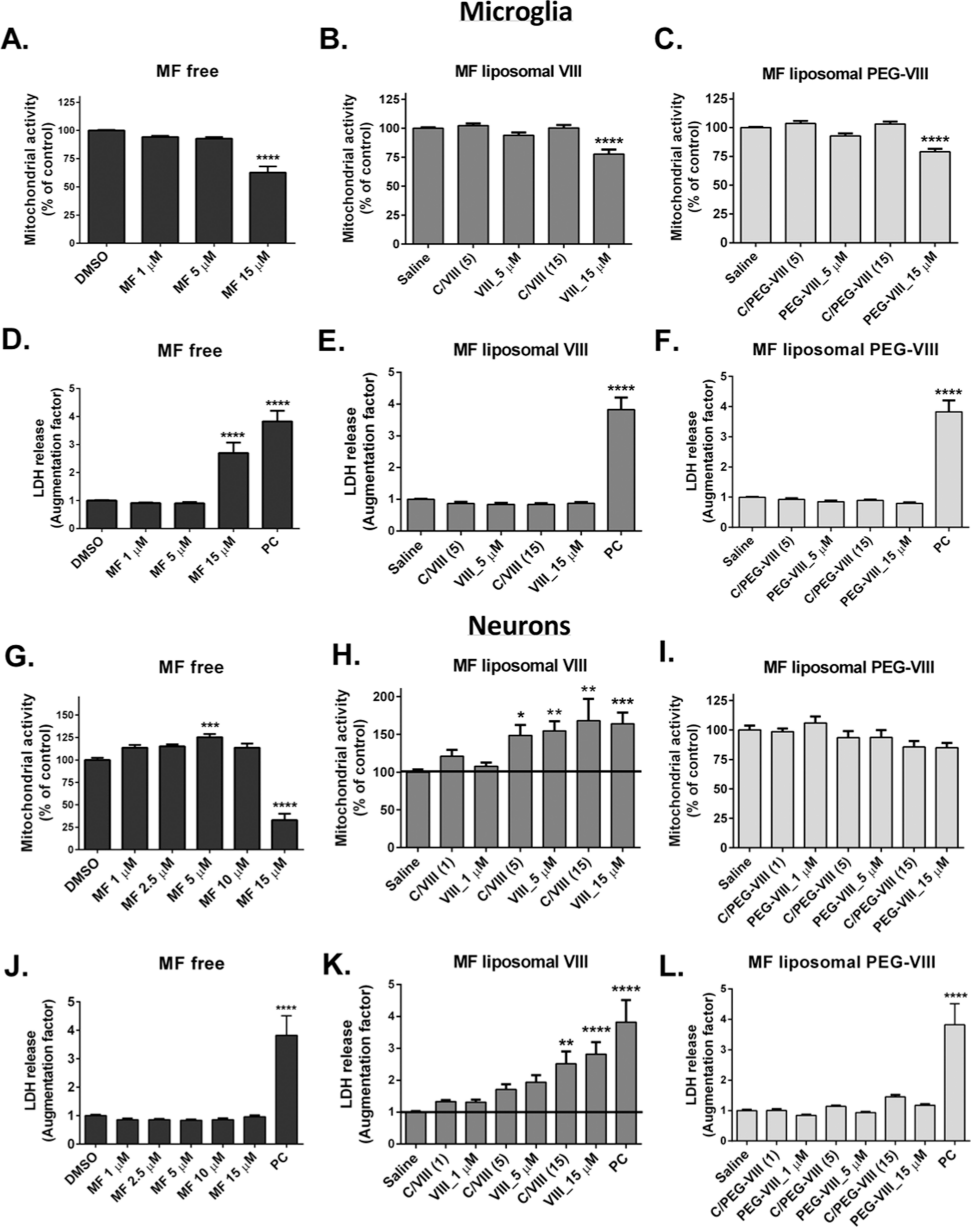
Effect of MF free and MF liposomal VIII and PEG-VIII on mitochondrial activity and LDH release of BV-2 microglia and primary cortical neurons. (A–C) Effect of MF free (1–15 μM), MF liposomal **VIII** and **PEG-VIII** (5–15 μM), and their respective controls (**C/VIII** and **C/PEG-VIII**) on mitochondrial activity of BV-2 cells compared to the respective vehicle. (D–F) Effect of MF free (1–15 μM), MF liposomal **VIII** and **PEG-VIII** (5–15 μM), and their respective controls (**C/VIII** and **C/PEG-VIII**) on LDH release in BV-2 cells. (G–I) Effect of MF free and MF liposomal **VIII** and **PEG-VIII** (1–15 μM), together with their respective controls (**C/VIII** and **C/PEG-VIII**) on the mitochondrial activity of cortical neurons compared to the respective vehicle. (J–L) Effect of MF free and MF liposomal **VIII** and **PEG-VIII** (1–15 μM), and their respective controls (**C/VIII** and **C/PEG-VIII**) on LDH release in cortical neurons. PC = positive control (Triton 2%). *P < 0.05, **P < 0.01, ***P < 0.001, ****P < 0.0001 vs DMSO/saline. Mean ± SEM, *N* = 3, measured in sextuplicates, evaluated by one-way ANOVA with Bonferroni’s post hoc test.

To assess whether an anti-inflammatory effect of MF liposomal could be safely achieved in a concentration range similar to that efficient in monocytes, we first evaluated the dose–response effect of MF free and MF liposomal VIII and PEG-VIII on mitochondrial activity (Figure 5A–C), reflecting energy homeostasis and LDH release (Figure 5D–F) as a marker of cellular damage of BV-2 mouse microglia. The dose of 15 μM of MF free estimated to present toxicity risks on monocyte and BV-2 cells altered the mitochondrial activity (Figure 5A) and induced LDH release indicating BV-2 cellular stress (Figure 5D). In contrast to free MF, both MF liposomal formulations **VIII** and **PEG-VIII** (15 μM) impacted the mitochondrial activity of BV-2 cells (Figure 5B,C) without inducing LDH release (Figure 5E,F), lower doses had no negative effect.^45^

While the role of microglia is to maintain homeostasis, neurons are responsible for processing and transmitting information via action potentials and neurotransmitters. They are electrically excitable due to the maintenance of a voltage gradient across the cell membrane. It was therefore necessary to determine whether the electric charge of liposomes could affect the proper neuronal functioning, and if so at what concentration. Mitochondrial activity is a crucial indicator of the neuronal metabolic rate and is essential for maintaining neuronal activity, the establishment of membrane excitability, and the execution of the complex processes of neurotransmission and plasticity.^51^ Thus, we evaluated the effect of MF free and MF liposomal **VIII** and **PEG-VIII** at MF concentrations ranging from 1 to 15 μM on mitochondrial activity (Figure 5G–I) and cellular stress/LDH release (Figure 5J–L) in primary mouse cortical neurons. Similar to microglia, free MF (15 μM) rapidly dampened the mitochondrial activity of neurons (Figure 5G) but without triggering the LDH release. MF free in the range from 1 to 10 μM moderately reduced neuronal LDH release by 14–16% (Figure 5J). The lowered basal LDH was accompanied by a slight increase of metabolically active cells treated with MF free (1–10 μM) (Figure 5G). Together with lowered LDH amounts, this result could indicate an improved native state and survival of the neuronal culture in comparison with the vehicle-treated cultures. In contrast, MF liposomal formulation **VIII** at 1 μM concentration had no effect on the metabolic activity of neurons (Figure 5H) and their cellular integrity (Figure 5K). Surprisingly, while the effect of MF liposomal **VIII** at 5 and 15 μM concentrations was not different from their respective liposomal controls **C/VIII**, they markedly increased neuronal metabolic activity up to 160% compared to saline (Figure 5H) and displayed deleterious effects on neuronal cultures, affecting neuronal integrity and causing cellular damage (Figure 5K).^45^ These unforeseen results may be due to the used amounts of positively charged phospholipids. Azzazy et al. observed that the use of cationic phospholipids below 150 μM of total lipids should maintain normal electrical activity without damage to neurons, but higher concentrations could trigger major alterations in the electrical activity of neurons affecting their electrophysiological behavior.^52^ This could explain our observations and indicate that the use of higher concentrations of cationic C/VIII phospholipids should be avoided for applications in neuronal cells. This is also in accord with previous studies and reviews reporting the harmful effects of some cationic liposomes.^10,53^ Cytotoxicity of cationic liposomes should be determined in a charge-dependent manner for a wide range of cells, mostly when increasing the ζ-potential of liposomes beyond ∼30 mV.^10^

The situation was different for pegylated liposomes **C/PEG-VIII** because PEG effectively covers a positive charge of liposomes (their charge was 0.2 mV compared to 27.9 mV for **C/VIII**). Indeed, within a concentration range of 1–15 μM, **PEG-VIII** did not alter the mitochondrial activity of neurons (Figure 5I) nor induced LDH elevation (Figure 5L). Similarly, Filion and Phillips observed in their study that the incorporation of 10 mol % 1,2-dipalmitoyl-*sn*-glycero-3-phosphoethanolamine (DPPE, 16:0 PE)-PEG 2000 abolished the toxicity of cationic vectors on murine macrophages.^54^ Thus, **PEG-VIII** formulation confirmed its advantage to be safe for neuronal cultures up to a 15 μM concentration of incorporated MF and was therefore selected for evaluation of the cellular uptake by BV-2 microglia and protective and anti-inflammatory effects at the CNS level.

### Uptake of MF Liposomal **PEG-VIII** by BV-2 Microglia

**Figure 6:**
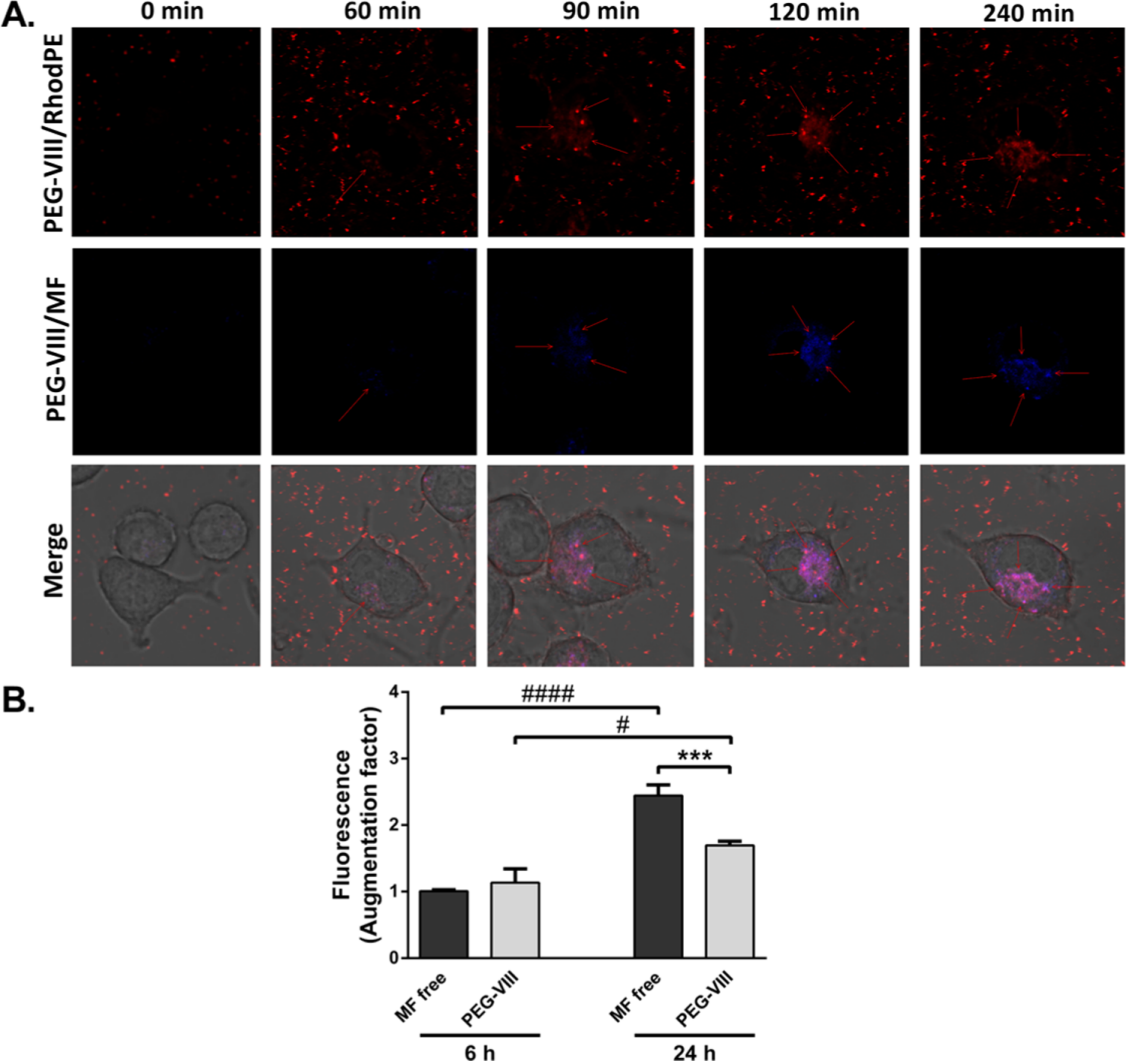
Uptake of MF liposomal **PEG-VIII** by BV-2 microglia. (A) BV-2 microglia uptake of **PEG-VIII** directly after treatment (*t* = 0 min), 60, 90, 120, and 240 min later. Red-**PEG-VIII** liposomes stained with Liss Rhod PE and blue-MF in PEG-VIII liposomes. (B) Comparison of incorporation and cell uptake based on fluorescence intensity of MF free and **PEG-VIII** in BV-2 cell lysates at 6 and 24 h after treatment. ***P < 0.001 MF free vs **PEG-VIII**, ^#^*P* < 0.05, ^####^*P* < 0.0001 MF free/PEG-VIII 6 h vs 24 h. Mean ± SEM, *N* = 3, measured in sextuplicates, evaluated by ANOVA with Bonferroni’s post hoc test.

Since the delivery capacity of **PEG-VIII** relies on its cellular uptake, as we have shown on THP-1-XBlue-MD2-CD14 human monocytes, we evaluated its cellular uptake by BV-2 mouse microglia. Using live cell imaging, we observed that the microglial uptake of **PEG-VIII** liposomes with incorporated Liss Rhod PE (**PEG-VIII**/Rhod PE) and the amount of MF liposomal (**PEG-VIII**/MF) increased in cells in a time-dependent manner. While after 60 min of treatment with PEG-VIII, MF can be only barely observed in cells, after 90 min and later, MF in **PEG-VIII** liposomes can be easily identified in BV-2 microglia (Figure 6A). This confirmed that PEG-VIII is internalized in microglia within the first 2 h after treatment. By measurement of MF fluorescence from cell lysates at later time points, we also determined that the amount of PEG-VIII (5 μM) in cells is similar to this MF free 5 μM at 6 h after treatment but lowered by 30% at 24 h (Figure 6B). Similarly, it has been reported that the inclusion of DPPE-PEG into liposomes can increase the viability of cells but also dampen liposomal uptake by macrophages.^55^ We speculate that this result could be attributed predominantly to different mechanisms of MF free vs **PEG-VIII** transport into cells. We showed that MF free passes into the model of lipid bilayer passively within a short time, and this mechanism could be applicable also for cell membranes, while **PEG-VIII** could be actively phagocytosed by microglia. However, the mechanisms of MF and **PEG-VIII** entry into cells need to be studied more in detail.

### Pretreatment with MF Liposomal Reduces LDH and TNF-α Release in BV-2 Microglia

**Figure 7:**
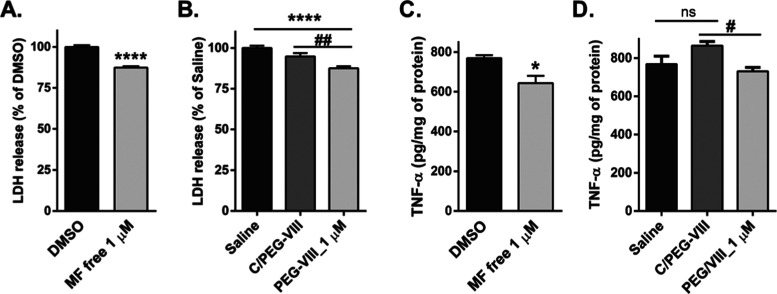
Protective and anti-inflammatory effects of **PEG-VIII** in BV-2 cells. BV-2 cells were pretreated for 1 h with MF free (1 μM), **PEG-VIII** (1 μM), and its respective control **C/PEG-VIII** before LPS (1 μg mL^–1^) stimulation. (A) Effect of free MF on LDH release at 24 h. (B) Effect of **PEG-VIII** and **C/PEG-VIII** on LDH release was studied at 24 h. Mean ± SEM, *N* = 4, measured in sextuplicates. (C) Effect of free MF on TNF-α release at 24 h. (D) Effect of PEG-VIII and C/PEG-VIII on TNF-α release at 24 h. *P < 0.05, ****P < 0.0001 vs respective vehicle, ^#^*P* < 0.05, ^##^*P* < 0.01 **PEG-VIII** vs **C/PEG-VIII**. Mean ± SEM, *N* = 4. Student’s *t* test (graphs A and C), one-way ANOVA with Bonferroni’s post hoc test (graphs B and D).

Knowing that **PEG-VIII** could deliver MF in microglial cells and 1 μM free MF exerts an anti-inflammatory effect,^24^ we investigated the cytoprotective and anti-inflammatory properties of MF integrated into **PEG-VIII**. We thus evaluated the effect of the concentration of **PEG-VIII** (1 μM) against LPS-induced inflammation in BV-2 cells. At this concentration, pretreatment with MF free reduced the LPS-stimulated LDH release by approximately 20% relative to the vehicle (Figure 7A).^45^ While **C/PEG-VIII** slightly reduced the LPS-triggered LDH release, the protective effect of **PEG-VIII** was similar to that of MF free (Figure 7B). This suggests that MF cytoprotective effect against LPS is retained in **PEG-VIII**.

We next investigated the anti-inflammatory potential of MF liposomal by analyzing the release of proinflammatory TNF-α protein from LPS-challenged microglia. MF liposomal **PEG-VIII** at a 1 μM concentration effectively reduced TNF-α levels related to the respective control **C/PEG-VIII** (Figure 7D). This inhibitory effect was comparable to that of MF free (1 μM) (Figure 7C). These results indicated that the encapsulation of MF does not alter its protective and anti-inflammatory potential while providing it with an estimated improved bioefficacy and bioavailability. This shows that **PEG-VIII** is an efficient therapeutic vector maintaining the anti-inflammatory properties of MF. Introducing cholesterol in the lipid composition of formulation **PEG-VIII** might have contributed to the beneficial effects of this formulation as cholesterol and lipid composition of membranes have been described as important factors determining the stability of bilayers, drug encapsulation efficiency and retention, and *in vivo* fate (tissue distribution and plasma clearance). Cholesterol plays a substantial role in the fluidity, stability, and permeability of lipid membranes.^56^ Altogether, these results arouse interest in the potential development of liposomal MF as a dietary supplement, as it has been already done for the most studied RSV or other polyphenols such as curcumin where encapsulation into nanoparticles showed improved solubility, stability, and bioavailability.^57−61^ Such a nutraceutical would be aimed at the prevention of any type of systemic and/or CNS inflammatory disease, preventing chronic disorders, maintaining or improving organ function, or even delaying the process of aging.^62,63^ The described liposomal formulation could find usefulness for the encapsulation of other promising stilbene molecules facing poor water solubility and promote their bioefficacy.

## Conclusions

In this study, we defined optimal liposomal composition and way of encapsulation of insoluble or poorly soluble stilbenoid compound macasiamenene F (MF) to enhance its cytoprotective and anti-inflammatory potential. From several compositions with different electrokinetic potentials, we identified PEG-VIII as the most interesting form based on its safety on THP-1 and THP-1-XBlue-MD2-CD14 human monocytes, BV-2 mouse microglia, and primary mouse cortical neurons. Moreover, PEG-VIII preserved the protective and anti-inflammatory potential of unencapsulated/free MF against LPS-induced peripheral inflammation and neuroinflammation. Encapsulation of MF increases its solubility and estimated bioefficacy and represents an appropriate form for *in vivo* administration.

## Methods

### Preparation of Liposomes

Nine different liposomal formulations (Table 1) were prepared using the following lipids: cholesterol (CHOL), 1,2-dioleoyl-*sn*-glycero-3-ethylphosphocholine (chloride salt) (EPC), cationic 3β-[*N*-(*N*′,*N*′-dimethylaminoethane)-carbamoyl]cholesterol hydrochloride (DC-CHOL), anionic 1-palmitoyl-2-oleoyl-*sn*-glycero-3-phospho-(1′-*rac*-glycerol) (sodium salt) (POPG), and 1,2-dioleoyl-*sn*-glycero-3-phosphoethanolamine-*N*-(lissamine rhodamine B sulfonyl) (ammonium salt) (18:1 Liss Rhod PE). All lipids were purchased from Avanti Polar Lipids (Alabaster, AL) with the exception of 1,2-distearoyl-*sn*-glycero-3-phosphoethanolamine-*N*-[amino(polyethylene glycol)-2000] (DSPE-PEG 2000; NOF Corporation, Japan). Lipid components with MF (experimental samples) or without (reference liposomal controls) were dissolved in chloroform (p.a., 99.8%, Sigma-Aldrich, Czech Republic). The organic solvent was removed by using the rotary vacuum evaporator with an elevated temperature above the highest transition temperature of the lipid in the mixture, followed by rehydration of the lipid film in an aqueous solvent (0.9% NaCl) and sonication. Liposomes were homogenized by manual membrane extrusion through a 100 nm polycarbonate filter (Whatman Nuclepore, Sigma-Aldrich, Czech Republic) using a manual extruder (LiposoFast, Avestin, Canada) 21 times. The size, polydispersity, and stability were determined using the dynamic light scattering (DLS) technique (Zetasizer Nano ZSP, Malvern, U.K.) in a low volume, quartz batch cuvette ZEN2112 (Malvern Panalytical Ltd., U.K.). The liposomes size and distribution were measured at a detection angle of 173° and temperature of 25 °C. The accumulation time was automatically determined during each measurement. The electrokinetic potential in colloidal dispersions of liposomes was determined as the value of the ζ-potential (Zetasizer Nano ZSP, U.K.). The morphology of liposomes was determined using transmission electron microscopy (TEM, Philips 208S Morgagni, FEI, Czech Republic) at a magnification ranging from 18,000× to 140,000× and an accelerating voltage of 80 kV. The content of MF in liposomes represented five molar percent, predetermined as a stable composition meeting requirements for size and homogeneity (Table S1). The stock solutions of liposomes were prepared at a concentration of MF 1 mM and then diluted in saline for desired concentrations used in dose–response studies. Control groups represented liposomal formulations alone without MF, used at the same concentration of lipids as the tested MF liposomal. The stability of liposomes was verified once per week using Zetasizer Nano during the period of biological activity testing.^45,64^

### Transition of MF into the Model Lipid Membrane and Uptake of Liposomes by Cells

The encapsulation of MF into liposomes was verified by spectrofluorometric measurements (spectrofluorometer Chronos DFD, ISS) and confocal microscopy (Leica TCS SP8MP, Leica Microsystems, Germany) based on autofluorescence properties of MF (Figures S5 and S6). The excitation and emission spectra of MF in 96% ethanol were measured using Chronos DFD, ISS, and Vinci software. Transition of MF 2 μM in water into the model of lipid membrane (EPC, 38 μM) was assessed directly (*t* = 0) after mixing both aqueous dispersions by spectrofluorometric measurements. The cellular uptake of MF liposomal (1 μM) by THP-1-XBlue-MD2-CD14 cells was observed at 24 h, and the uptake of **PEG-VIII** (10 μM) with incorporated 18:1 Liss Rhod PE (0.1 mol %) by BV-2 cells was observed immediately (*t* = 0), 60, 90, 120, and 240 min after treatment using super-resolution live cell imaging (Leica TCS SP8MP, Leica Microsystems, Germany).

### Animals

Female C57BL/6JRj mice (Janvier, France) were housed under controlled conditions according to the FELASA guidelines and recommendations at 22 °C with a 12 h light–dark cycle and free access to water and food. All animal care and use were performed in accordance with the policies of the European Community Directive 86/609/EEC. *Ex vivo* experiments were approved by the local ethical committee. Every effort was made to minimize the number of animals used in this study.

### Cell Culture and Differentiation

The THP-1 human monocytic leukemia cell line was purchased from the ECACC (Salisbury, U.K.), THP-1-XBlue-MD2-CD14 from Invivogen (San Diego, CA), and BV-2 murine microglia were generated from primary microglial culture by infection with *v-raf*/*v-myc* oncogene carrying J2 retrovirus.^65^ All cell lines were cultured as previously described.^24^ All experiments on cell lines were performed in a serum-free medium.

### *Ex Vivo* Culture of Mouse Cortical Neurons

Cortical neurons were isolated from 14-day-old embryos of pregnant C57BL/6JRj mice (Janvier, France). Brains and cortices were dissected, and the olfactory bulbs and meninges were removed. The cortices cut into small pieces were dissociated in gentleMACS tube C (Miltenyi Biotec) using a Neural Tissue Dissociation Kit (P) (Miltenyi Biotec) according to the manufacturer’s protocol. The dissociation was carried out using a gentleMACS Dissociator (Miltenyi Biotec). The dissociated tissue was filtered through 40 μm cell strainers (BD Biosciences). Neurons were cultured in Neurobasal medium, supplemented with GlutaMAX 0.5 mM (Gibco), B-27 supplement (Gibco), and antibiotics [100 U mL^–1^ penicillin and 100 μg mL^–1^ streptomycin (Biosera, France)] in 24-well Corning poly-d-lysine BioCoat plates (Corning, MA) at a concentration of 2.5 × 10^5^ cells/well. The next day, neuronal cultures were treated with 5-fluoro-2′-deoxyuridine (2 μM FdU, Sigma) and uridine (2 μM U, Sigma) to suppress the proliferation of other nonneuronal cells potentially occurring in the cell culture. Every 3rd day, half of the medium was replaced with fresh medium. Cortical neurons were cultured for 12 days and then used for experiments.^66^

### Cell Viability Dose–Response Study

Undifferentiated floating THP-1 and THP-1-XBlue-MD2-CD14 cells in serum-free RPMI 1640 medium were seeded into 96-well plates (5 × 10^4^ cells/well). After 2 h, MF free, MF liposomal, and respective lipid controls were added at concentrations ranging from 0.125 to 15 μM. The cell viability was assessed 24 h later using a Cell Proliferation Reagent WST-1 kit (Roche Diagnostics, Basel, Switzerland). Based on the resulting viability curves, the IC_50_ values were calculated according to four-parameter logistic (4PL) analysis. The LDH release was determined after 24 h using a Cytotoxicity Detection Kit (Roche, France). In the case of adherent BV-2 cells, the dose–response assays were carried out in the exponential phase of growth established by a pilot study in 24-well plates. Briefly, BV-2 cells were seeded in the evening in a complete DMEM medium at a density of 1.5 × 10^5^ cells/well, washed with PBS the next day, and the medium was replaced by a serum-free medium. Cells were treated for 24 h with MF free and MF liposomal **VIII** and **PEG-VIII** at concentrations ranging from 1 to 15 μM. 24 h later, mitochondrial activity was evaluated using Cell Titer 96 Aqueous One Solution Cell Proliferation Assay (MTS; Promega, France). Cytotoxicity was assessed using the Cytotoxicity Detection Kit (Roche, France) and presented as the percentage of lactate dehydrogenase (LDH) released by damaged cells compared to vehicle-treated cells. Same reagents were used to assess the effect of compounds on mitochondrial activity and LDH release in cortical neurons in culture.

### Determination of the NF-κB/AP-1 Activity

The activity of NF-κB/AP-1 was measured on THP-1-XBlue-MD2-CD14 cells (Invivogen, CA). This cell line stably expresses the NF-κB and AP-1 inducible secreted embryonic alkaline phosphatase (SEAP) reporter gene, the activity of which was detected using a QUANTI-Blue reagent (Invivogen, San Diego). Cells in serum-free medium RPMI 1640 medium (5 × 10^4^ cells/well, 96-well plate) were incubated for 2 h at 37 °C. Compounds MF free and MF liposomal **I–VIII** were added at a concentration of 1 μM, together with **C/I–VIII** and respective vehicles. DMSO (0.1% (v/v)) was used as a vehicle for MF free and saline (0.9% NaCl) for MF liposomal **I–VIII** and **C/I–VIII**. After 1 h of incubation, the cells were stimulated with LPS (1 μg mL^–1^),^67^ and the activity of NF-κB/AP-1 was evaluated after 24 h. Spectrophotometric measurements at 655 nm were carried out in a Fluostar Omega Microplate Reader (BMG Labtech, Ortenberg, Germany).

### Detection of LDH Release and Proinflammatory TNF-α Expression

The LDH release and protein expression of TNF-α in BV-2 cells pretreated for 1 h with MF free (1 μM), **PEG-VIII** (1 μM), **C/PEG-VIII**, and respective vehicles were determined 24 h after LPS (1 μg mL^–1^) stimulation^68^ from supernatants using Cytotoxicity Detection Kit (Roche, France) and AlphaLISA mTNFα (AL505C) kit (PerkinElmer, MA) according to manufacturers’ protocols. The total quantities of TNF-α cytokine produced were calculated and normalized to the amounts of total protein determined by the Bradford protein assay (BioRad, France).

### Statistical Analysis

Statistical analysis was carried out using GraphPad Prism 6.01 software (San Diego, CA). Data were expressed as the mean ± SEM. The IC_50_ values for resultant viability curves were calculated using four-parameter logistic (4PL) analysis. Statistical analyses of differences between groups were performed using the parametric Student’s *t* test (for comparison of two groups) and one-way ANOVA with Bonferroni’s post hoc test (for more than two groups). Values of *P* less than 0.05 were considered statistically significant.

## Supporting Information Available

Selection of MF molar content in liposomes; cell viability and calculated IC_50_ values of MF free and MF liposomal; DLS distributions of the anionic, neutral, cationic, and PEGylated nanoliposomes; excitation and emission fluorescent spectra of MF; comparison of emission spectra of MF liposomal formulations with MF free; spectrophotometric evaluation of the MF transition into the lipid bilayer and the uptake of MF liposomal by THP-1-XBlue-MD2-CD14 human monocytes; and effect of cationic MF liposomal formulations and their reference controls on LDH release from THP-1 and THP-1-XBlue-MD2-CD14 human monocytes (PDF)

## Abbreviations

AD: Alzheimer’s disease
AP-1: activator protein 1
BBB: blood–brain barrier
CHOL: cholesterol
DAMPs: damage-associated molecular patterns
DC-CHOL: 3β-[*N*-(*N*′,*N*′-dimethylaminoethane)-carbamoyl]cholesterol hydrochloride
DSPE-PEG 2000: 1,2-distearoyl-*sn*-glycero-3-phosphoethanolamine-*N*-[amino(polyethylene glycol)-2000]
EPC: 1,2-dioleoyl-*sn*-glycero-3-ethylphosphocholine (chloride salt)
18:1 Liss Rhod PE: 1,2-dioleoyl-*sn*-glycero-3-phosphoethanolamine-*N*-(lissamine rhodamine B sulfonyl) (ammonium salt)
IκB: inhibitor of NF-kappa B transcription factor
IL-1β: interleukin 1β
LDH: lactate dehydrogenase
LPS: lipopolysaccharide
MCAo: middle cerebral artery occlusion
MF: macasiamenene F
NF-κB: nuclear factor kappa-light-chain enhancer of activated B-cells
PAMPs: pathogen-associated molecular patterns
PD: Parkinson’s disease
PEG: poly(ethylene glycol)
POPG: 1-palmitoyl-2-oleoyl-*sn*-glycero-3-phospho-(1′-*rac*-glycerol) (sodium salt)
RSV: resveratrol
SEAP: secreted embryonic alkaline phosphatase
TLR4: toll-like receptor 4
TNF-α: tumor necrosis factor α
