# Supplementary material for: Enhancing Solubility and Bioefficacy of Stilbenes by Liposomal Encapsulation—The Case of Macasiamenene F
Source: ACS Omega. 2024 Feb 15;9(8):9027–39. doi: 10.1021/acsomega.3c07380 (PMC10905713; doi:10.1021/acsomega.3c07380)
Supplement: Supplementary file 1 — ao3c07380_si_001.pdf [file ao3c07380_si_001.pdf]

Supporting Information for

Enhancing Solubility and Bioefficacy of Stilbenes by Liposomal Encapsulation – The Case of Macasiamenene F

Veronika Brezani[a,b,c,*], Nicolas Blondeau[c], Jan Kotouček[b], Eva Klásková[b,d], Karel Šmejkal[e], Jan Hošek[a,b], Eliška Mašková[b], Pavel Kulich[b], Vilailak Prachyawarakorn[f], Catherine Heurteaux[c,$] and Josef Mašek [b,$]

[a]Department of Molecular Pharmacy, Faculty of Pharmacy, Masaryk University, Palackého tř. 1946/1, CZ-612 00, Brno, Czech Republic;

[b]Department of Pharmacology and Toxicology, Veterinary Research Institute, Hudcova 296/70, CZ-621 00, Brno, Czech Republic;

[c]Université Côte d'Azur, CNRS, IPMC, UMR7275, 660 Route des Lucioles, Sophia Antipolis, F-06560, Valbonne, France;

[d]Department of Pharmacology, Faculty of Medicine, Masaryk University, Kamenice 753/5, CZ-625 00, Brno, Czech Republic;

[e]Department of Natural Drugs, Faculty of Pharmacy, Masaryk University, Palackého tř. 1946/1, CZ-612 00, Brno, Czech Republic;

[f]Chulabhorn Research Institute, Kamphaeng Phet 6 Road, Laksi, TH-10210, Bangkok, Thailand

[$] These authors share the last author position.

* Corresponding author.

Table S1: Selection of MF molar content in liposomes*. Different concentrations of MF (3, 5, or 7 mol %) were loaded into EPC liposomes, and the average size and PDI were determined using Zetasizer Nano ZSP. N=3. Mean±SD.*

**Sample**	**Composition (mol %)**		**Z-average (nm)**	**Polydispersity index (PDI)**
	**MF**	**EPC**		
1A	0	100	193.2±6	0.188±0.056
1B	3	97	184.5±9	0.150±0.021
1C	5	95	198.4±10	0.151±0.047
1D	7	93	206.2±4	0.199±0.063

Table. S2: Cell viability and calculated IC_{50} values of MF free and MF liposomal *24h after treatment of THP-1 and THP-1-XBlue-MD2-CD14 monocytes determined by WST-1 assay. N=3. Mean±SEM.*

	**IC_{50} (µM)**	
**Sample**	**THP-1**	**THP-1-XBlue-MD2-CD14**
**MF free**	5.7±1.1	5.4±1.3
**I**	>5	>5
**II**	>15	>15
**III**	>5	>5
**IV**	>15	>15
**V**	>5	>5
**VI**	>15	>15
**VII**	>5	>5
**VIII**	>5	>5

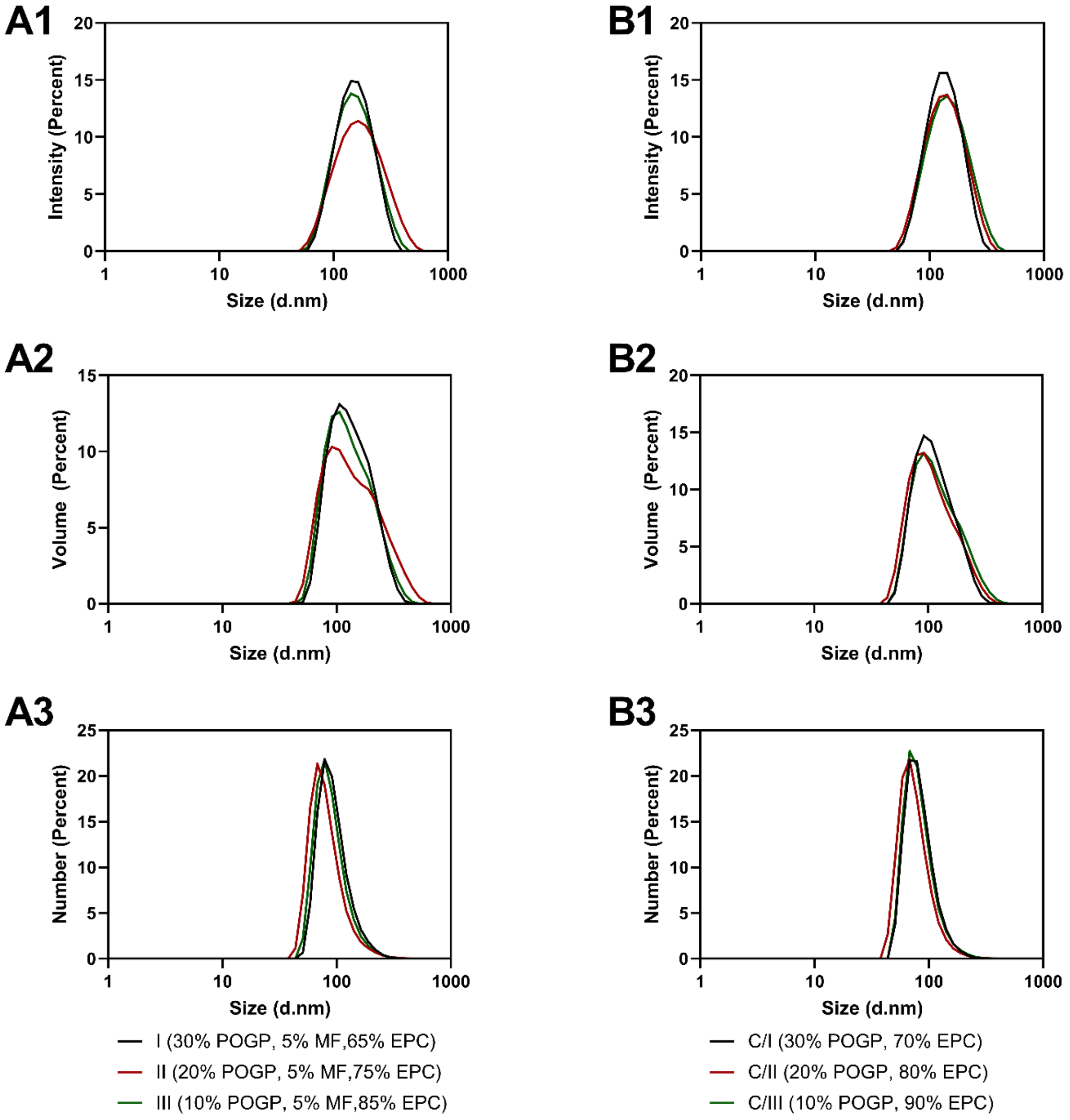

Fig. S1: DLS distributions of the anionic nanoliposomes. *The DLS analysis with index 1 represents the distribution by intensity; 2) by volume and index 3) shows the distribution by number. The index A) represents the liposomes with 5 mol % of macasiamenene F and B) their reference controls.*

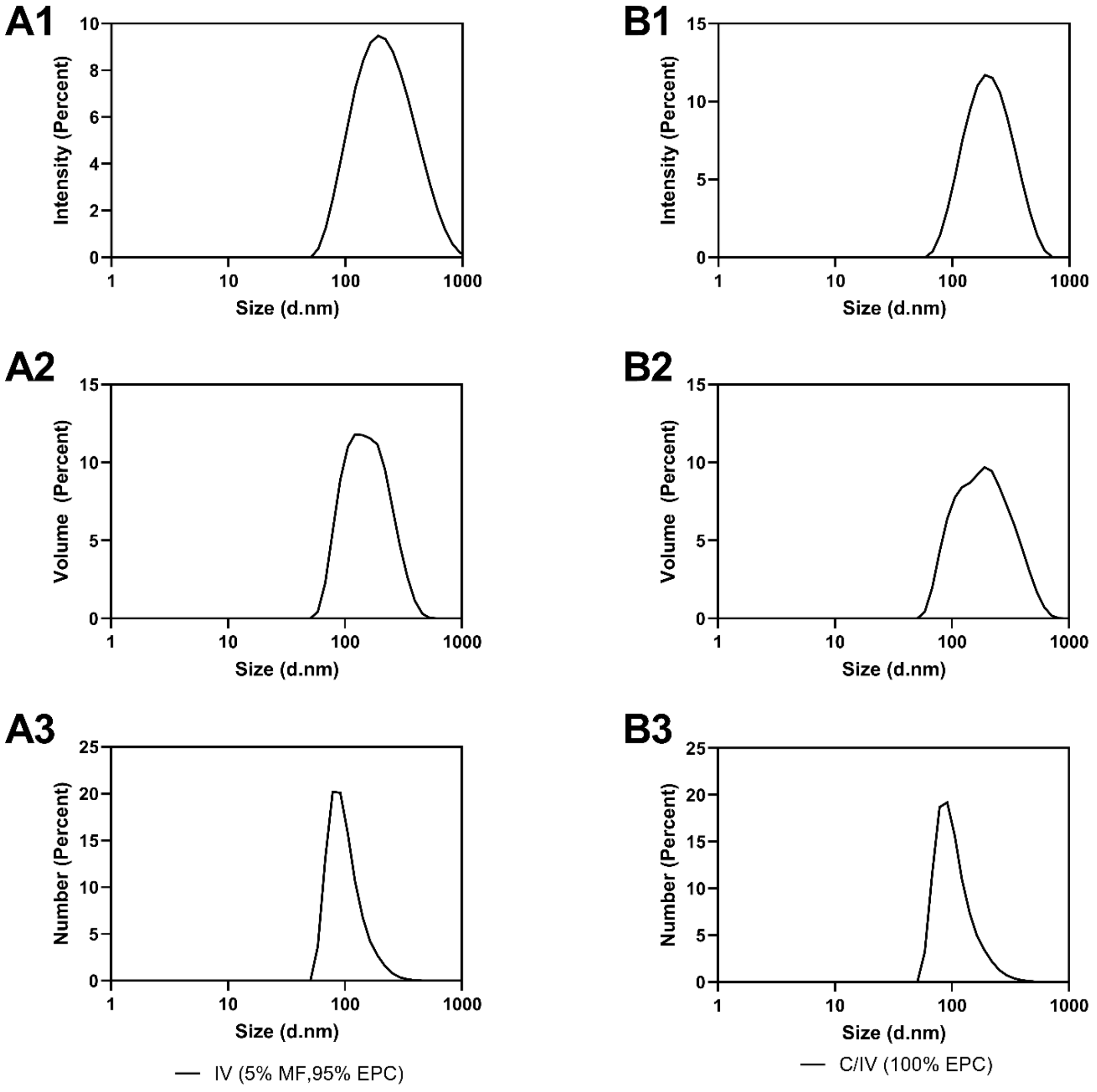

Fig. S2: DLS distributions of the neutral nanoliposomes. *The DLS analysis with index 1 represents the distribution by intensity; 2) by volume and index 3) shows the distribution by number. The index A) represents the liposomes with 5 mol % of macasiamenene F and B) their reference controls.*

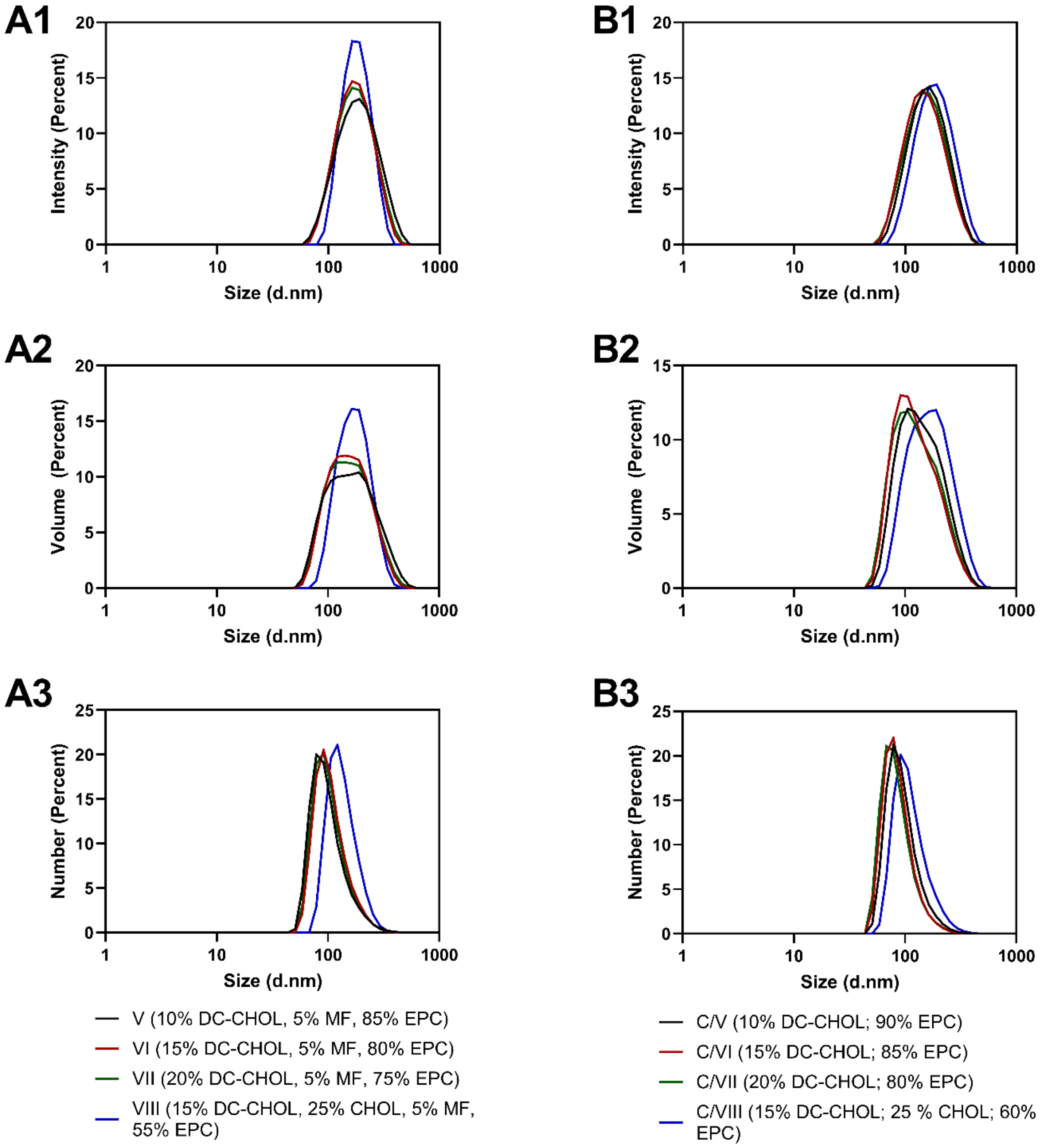

Fig. S3: DLS distributions of the cationic nanoliposomes. *The DLS analysis with index 1 represents the distribution by intensity; 2) by volume and index 3) shows the distribution by number. The index A) represents the liposomes with 5 mol % of macasiamenene F and B) their reference controls.*

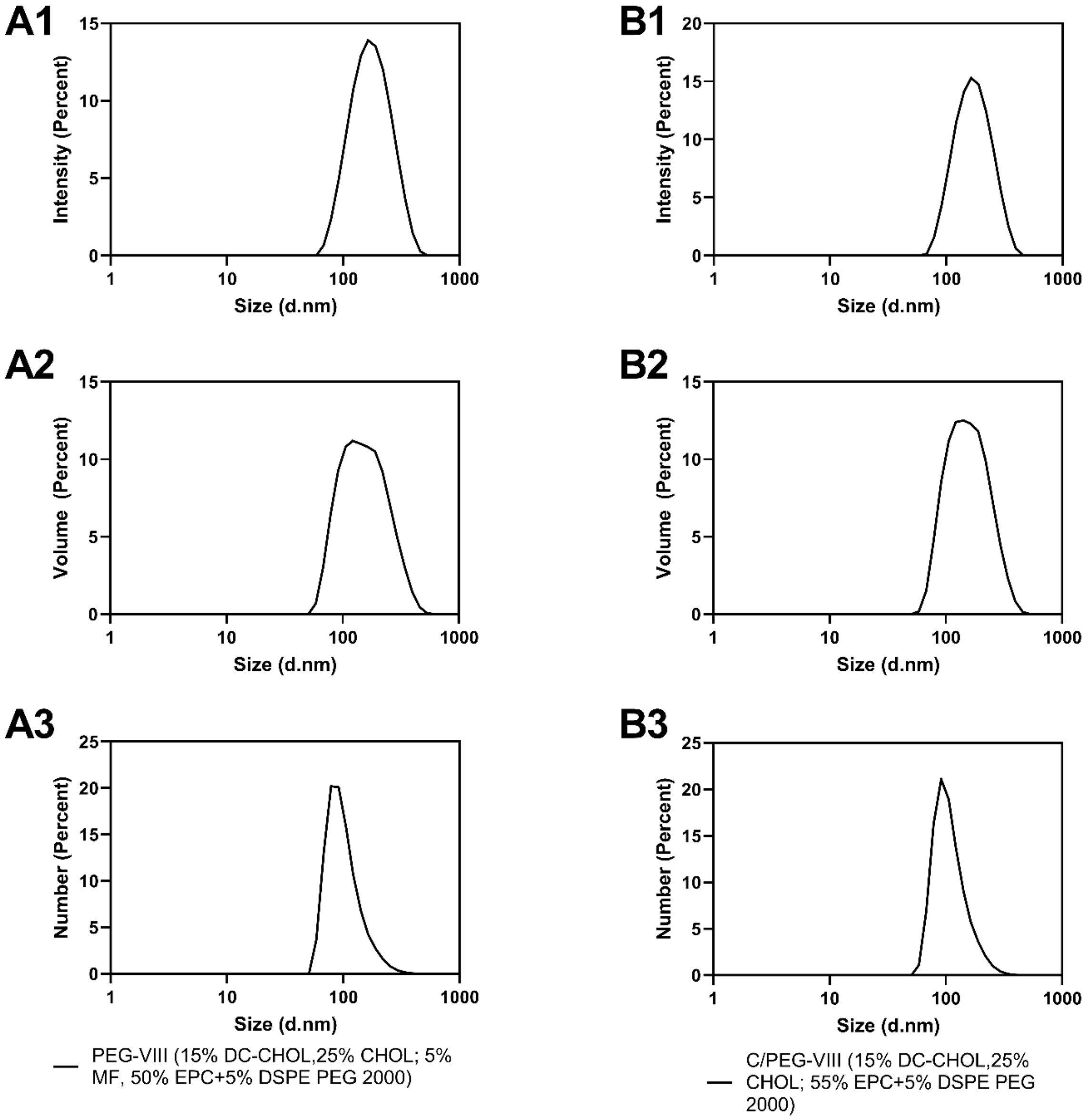

Fig. S4: DLS distributions of the PEGylated nanoliposomes. *The DLS analysis with index 1 represents the distribution by intensity; 2) by volume and index 3) shows the distribution by number. The index A) represents the liposomes with 5 mol % of macasiamenene F and B) their reference controls.*

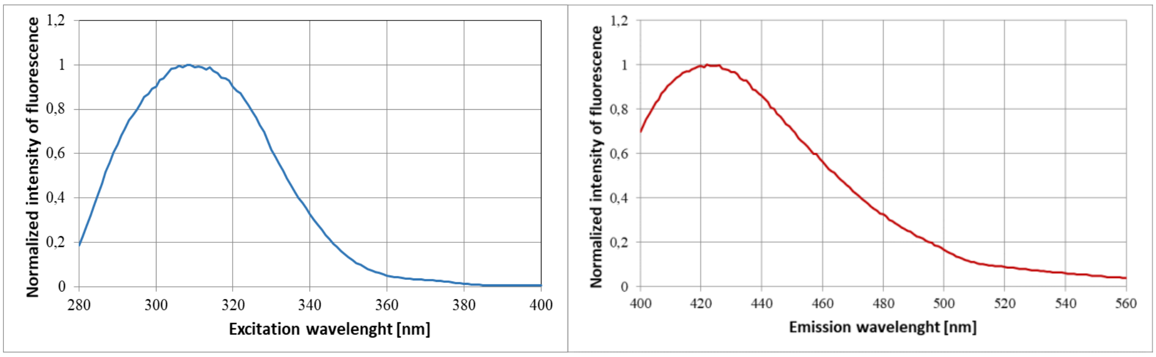

***Fig. S5: Excitation (left) and emission (right) fluorescent spectra of MF in 96% ethanol*.**

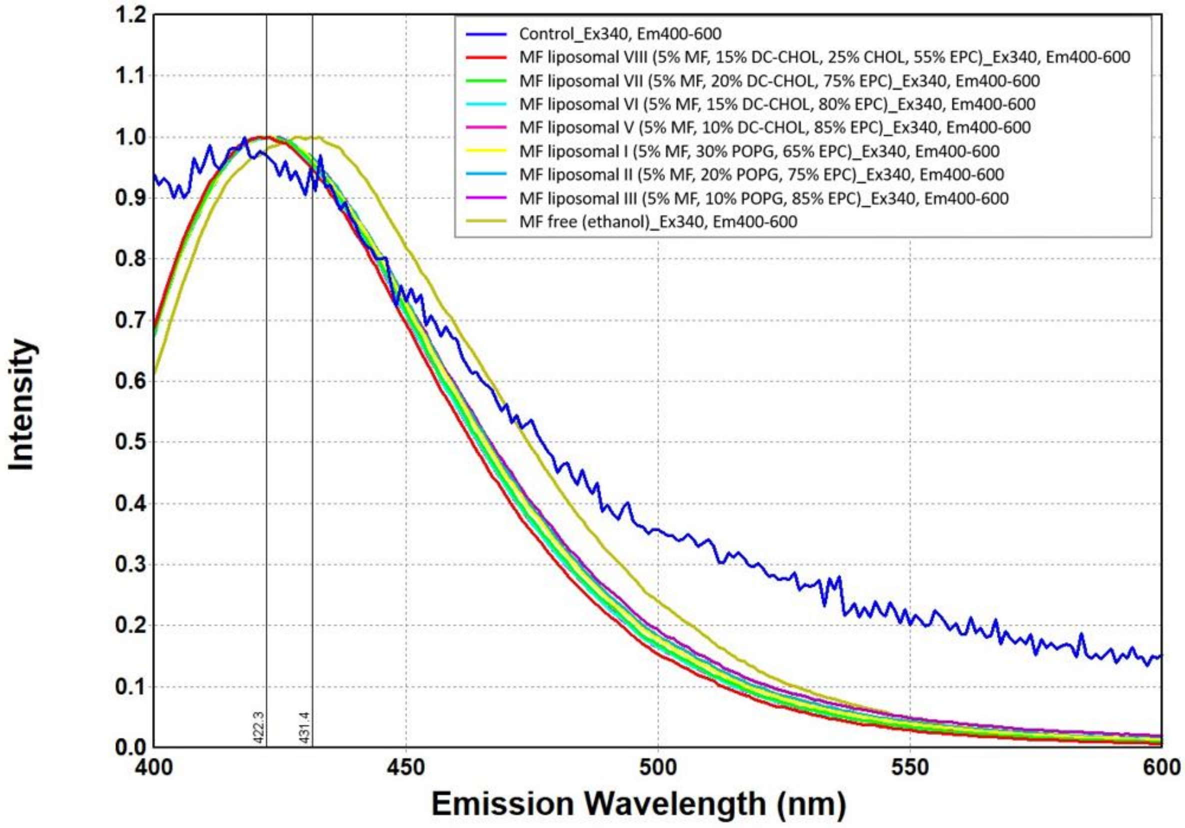

Fig. S6*: *Comparison of emission spectra of MF liposomal formulations with MF free *determined by spectrofluorometric measurement based on MF autofluorescence properties.*

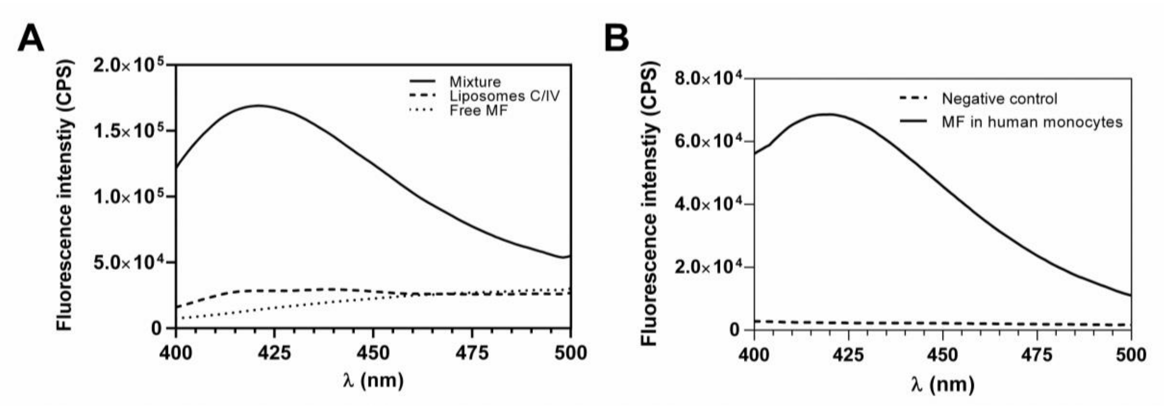

Fig. S7: Spectrophotometric evaluation of the MF transition into the lipid bilayer and the uptake of MF liposomal by THP-1-XBlue-MD2-CD14 human monocytes. ***A***, *Passage of MF free into the lipid membrane of EPC liposomes. Dotted line-MF free (2 μM) in water, dashed line-liposomes in water (EPC 38 μM), solid line-mixture of both at t=0.* ***B***, *The uptake of MF liposomal by THP-1-XBlue-MD2-CD14 human monocytes at 24 h. Solid line-MF fluorescence in human monocyte cell lysates. Dashed line-negative control.*

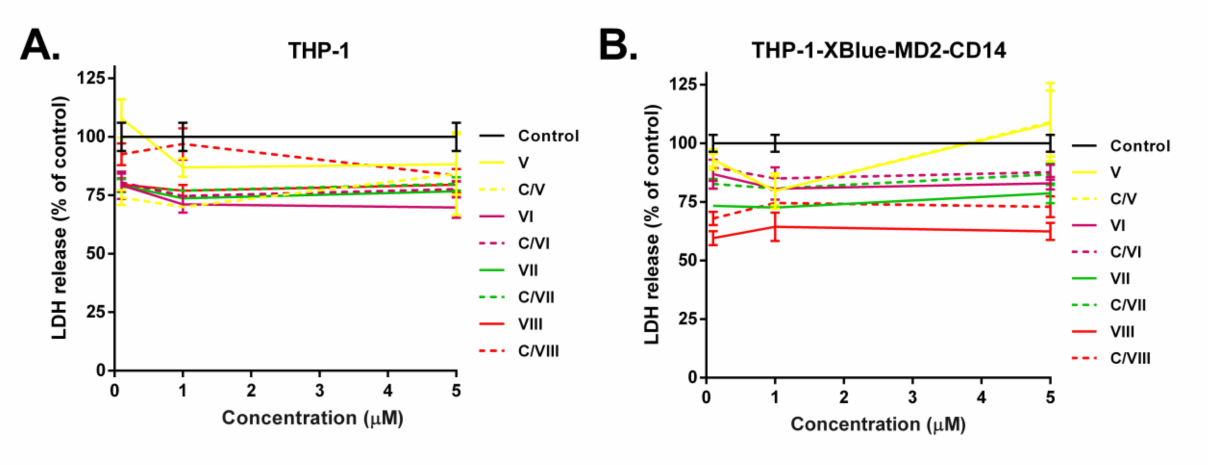

Fig. S8: The effect of cationic MF liposomal formulations and their reference controls on LDH release from THP-1 and THP-1-XBlue-MD2-CD14 human monocytes. *Determined at 24h after treatment, compared to non-treated control (spontaneous release). N=3. Mean±SEM.*
